# The Unexplored Wound Healing Activity of *Urtica dioica* L. Extract: An In Vitro and In Vivo Study

**DOI:** 10.3390/molecules26206248

**Published:** 2021-10-15

**Authors:** Athanasia I. Kasouni, Theodoros G. Chatzimitakos, Constantine D. Stalikas, Theoni Trangas, Alexandra Papoudou-Bai, Anastassios N. Troganis

**Affiliations:** 1 Laboratory of Biophysical Chemistry, Department of Biological Applications and Technologies, University of Ioannina, 45110 Ioannina, Greece; athanasia_kasouni@yahoo.gr; 2 Laboratory of Analytical Chemistry, Department of Chemistry, University of Ioannina, 45110 Ioannina, Greece; chatzimitakos@outlook.com; 3 Laboratory of Biochemistry, Department of Biological Applications and Technologies, University of Ioannina, 45110 Ioannina, Greece; ttrangas@uoi.gr; 4 Department of Pathology, Faculty of Medicine, School of Health Sciences, University of Ioannina, 45110 Ioannina, Greece; apapoudou@uoi.gr

**Keywords:** wound healing, histopathological study, cell proliferation and migration, cell cycle progression, anti-inflammatory, *Urtica dioica* L.

## Abstract

Wound healing is a great challenge in many health conditions, especially in non-healing conditions. The search for new wound healing agents continues unabated, as the use of growth factors is accompanied by several limitations. Medicinal plants have been used for a long time in would healing, despite the lack of scientific evidence veryfying their efficacy. Up to now, the number of reports about medicinal plants with wound healing properties is limited. *Urtica dioica* L. is a well-known plant, widely used in many applications. Reports regarding its wound healing potential are scant and sparse. In this study, the effect of an *Urtica dioica* L. extract (containing fewer antioxidant compounds compared to methanolic or hydroalcoholic extracts) on cell proliferation, the cell cycle, and migration were examined. Additionally, antioxidant and anti-inflammatory properties were examined. Finally, in vivo experiments were carried out on full-thickness wounds on Wistar rats. It was found that the extract increases the proliferation rate of HEK-293 and HaCaT cells up to 39% and 30% after 24 h, respectively, compared to control cells. The extract was found to increase the population of cells in the G_2_/M phase by almost 10%. Additionally, the extract caused a two-fold increase in the cell migration rate of both cell lines compared to control cells. Moreover, the extract was found to have anti-inflammatory properties and moderate antioxidant properties that augment its overall wound healing potential. Results from the in vivo experiments showed that wounds treated with an ointment of the extract healed in 9 days, while wounds not treated with the extract healed in 13 days. Histopathological examination of the wound tissue revealed, among other findings, that inflammation was significantly reduced compared to the control. *Urtica dioica* L. extract application results in faster wound healing, making the extract ideal for wound healing applications and a novel drug candidate for wound healing.

## 1. Introduction

A wound is a disruption of cellular and anatomical continuity of living tissue [1]. In wound treatment, a cascade of physiological actions takes place, which can be divided into four complex phases, implicating continual cell–cell and cell–matrix interactions. The stages involved in the healing process are hemostasis, inflammation, cellular proliferation, and tissue remodeling. Depending on the time needed to complete the wound healing process, wounds can be assorted into acute and chronic wounds. In both cases, inflammatory responses cause pain and swelling of the wound site, resulting in the discomfort of patients [2]. Moreover, untreated wounds are more susceptible to infections by microorganisms, which can potentially cause further health problems. Therefore, wound healing must be completed in the shortest possible time. To address this issue, among others, growth factors, including epidermal growth factor (EGF) and transforming growth factor (TGF), have been examined [1]. However, growth factor usage is accompanied by several limitations, such as a short half-life, high manufacturing costs, and difficulties with target site delivery [3]. Therefore, the search for new wound healing agents continues unabated. Medicinal plants are important for traditional medicine, which is becoming increasingly popular in many countries, owing to the low cost and high availability of plants [4,5]. There are quite a few reports dealing with the wound healing potential of various plants, which in many cases was attributed to their antioxidant and antimicrobial properties, while reports on the wound healing properties of plants, not attributed, only, to such properties are scanty and sparse [6,7,8,9]. Plant extracts that can produce different solvents, methanolic or alcoholic extracts commonly contain a higher number of antioxidant compounds, and therefore they are studied more extensively for wound healing applications [10,11,12,13,14].

*Urtica dioica* L. (commonly referred to as nettle or stinging nettle) is a herbaceous perennial flowering plant of the *Urticaceae* family that grows in Asia, America, North Africa, and Europe [15]. It has great economic potential due to its multi-utilitarian nature [16]. Recent studies have highlighted the pharmacological properties of *Urtica dioica* L., it being immunomodulatory, diuretic, anti-inflammatory, antioxidant, antimicrobial, antiulcer, analgesic and anti-allergic, owing to which extracts of the plant are used in many commercially available products [15,16]. So far, there are published reports highlighting the antioxidant, anti-inflammatory, and antibacterial properties of *Urtica dioica* L. extracts (mainly hydroalcoholic or methanolic extracts), rendering the plant a good candidate for wound healing applications [17,18,19]. 

The present study was undertaken to evaluate the wound healing potential of the aqueous extract (not deriving from direct boiling of the plant) of *Urtica dioica* L. The wound healing potential was evaluated in vitro by examining the effect of the extract on cell viability, the cell cycle, and cell migration. Next, the anti-inflammatory and antioxidant activity were evaluated, to gain a better insight into the side mechanisms that augment the overall wound healing effect. Finally, the wound healing potential was evaluated by examining the healing of full-thickness wounds on Wistar rats and a histopathological study was carried out on the wounds. The study was undertaken since the wound healing potential of an aqueous extract, poor in antioxidant compounds, has not been examined so far.

## 2. Results and Discussion

### 2.1. Extract Preparation

The extraction yield was 7 ± 1% (*w*/*w*) of the dry weight. This low percentage is attributed to the Soxhlet extraction preceding the boiling step. Owing to the Soxhlet step that precedes the main extraction step, the as-prepared aqueous extract differs from previously reported aqueous extracts of *Urtica dioica* L., prepared by direct boiling of the plant [20]. As a result, differences in its biological activity might arise. The above notion was validated from our experiments since the extract from the directly boiled plant exhibited no biological action (data not shown) compared to the proposed extract, as can be seen in the following sections.

### 2.2. Phytochemical Analysis

In order to limit down and gain further insight into the categories of compounds that are present, phytochemical screening experiments were carried out. Results showed that the extract contains the following classes of compounds: saponins, flavonoids, carbohydrates, ketoses, resins, and coumarins. Terpenoids, alkaloids, tannins, phlobatannins, phenols, steroids, xanthoproteins, proteins, amino acids, carboxylic acids, quinones, reducing sugars, monosaccharides, cardiac glycosides, anthraquinones, and aldoses were absent from the plant extract, examined herein. Some of the above classes of compounds have been correlated with the wound healing activity of various medicinal plants [21]. Although phytochemical analysis cannot come to a definite conclusion regarding compounds responsible for the effects, it can hint towards potential biological activity.

**Figure 1:**
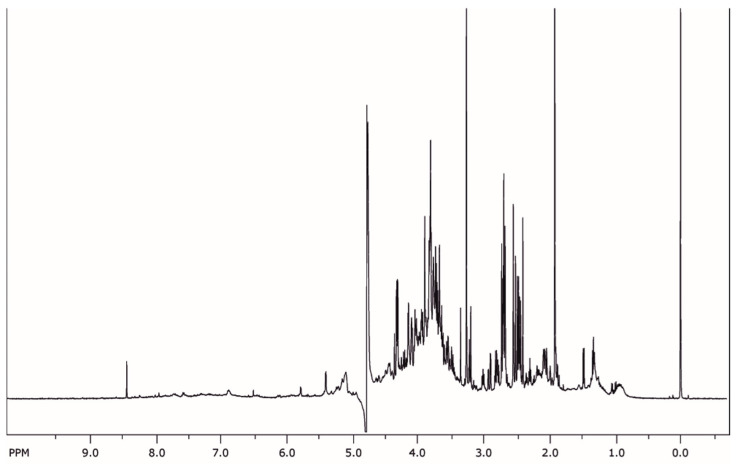
^1^H NMR fingerprint of the extract prepared from *Urtica dioica* L.

The NMR fingerprint of the extract can be seen in Figure 1. Between 6 and 8 ppm, only a few signals are visible, bespeaking that protons from aromatic compounds are limited, and therefore respective compounds are not in abundance in the extract. Additionally, multiple peaks can be seen in the region 2.5–4 ppm which can be attributed to saponins and hydrocarbons. Although more information cannot be derived from the NMR fingerprint, it serves as a valuable benchmark by which to assess the composition of the extracts obtained from different plants.

### 2.3. Cell Viability

**Figure 2:**
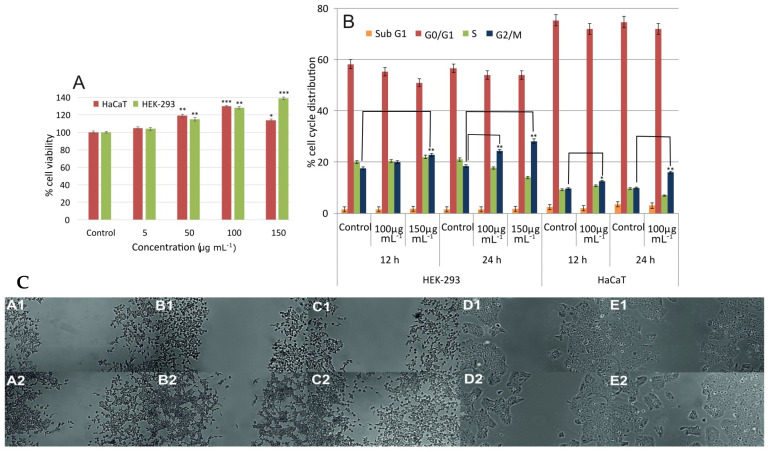
(**A**) Effect of different concentrations of the extract on the cell viability of HEK-293 and HaCaT cells, after 24 h of incubation (statistically significant differences for *p* < 0.05, *p* < 0.01, and *p* < 0.001 are denoted with *, **, and ***, respectively); (**B**): % cell cycle distribution of HEK-293 and HaCaT cells treated with 100 and 150 μg mL^−1^ of the extract after 12 and 24 h; (**C**) scratch assay photos of HEK-293 cells (A1: control 0 h, A2: control 24 h, B1: treated with 100 μg mL^−1^ at 0 h, B2: treated with 100 μg mL^−1^ at 24 h, C1: treated with 150 μg mL^−1^ at 0 h, and C2: treated with 150 μg mL^−1^ at 24 h) and HaCaT cells (D1: control 0 h, D2: control 24 h, E1: treated with 100 μg mL^−1^ at 0 h, and E2: treated with 100 μg mL^−1^ at 24 h).

Promoting cell proliferation is beneficial for the proliferation step of a wound healing process. Therefore, cell viability was examined in the presence and absence of the extract. For the in vitro study, HEK-293 and HaCaT cells were employed, based on previous studies, so as to examine the potential of the extract to be used for dermal and kidney injuries [22,23,24]. The viability of HEK-293 cells treated with the extract was increased compared to that of the untreated cells (control) after 24 h of incubation (Figure 2A). Maximum cell viability (i.e., 139 ± 2%) was achieved upon addition of 150 μg mL^−1^ of the extract. This value was statistically significant for *p* < 0.001. In the case of HaCaT cells (keratinocytes from human skin), cell viability was found to be increased by 30% upon incubation with 100 μg mL^−1^ of the extract for 24 h. Thus, this concentration was employed for further experiments since optimum results were obtained. Based on the above, the prepared extract is a good candidate for wound healing applications. Moreover, since the extract promotes cell proliferation on HEK-293 cells, it is a good candidate for the treatment of acute kidney injuries. On the contrary, the extract from directly boiling the plant does not increase cell viability, suggesting that there are constituents that hinter the increase in cell viability and that by employing our extraction method, such molecules are removed [25]. There are reports of plant extracts that promote wound healing in animal models, without increasing the viability of cells [26,27]. This effect was mainly attributed to the antioxidant compounds in the extracts. However, such a mechanism of action is only applicable to the first stages of wound healing, where there are plenty of free radicals [1]. Promoting cellular proliferation is another way to accelerate healing, and its role is crucial in the healing process [28].

### 2.4. Cell Cycle Progression

Cell cycle progression of the two cell lines, incubated in the absence and presence of the extract was examined by measuring the DNA content of the nuclei, using flow cytometry. The distribution of cells in the four phases (Figure 2B) shows that the extract increased the population of G_2_/M cells in a time-dependent manner, while it decreased the percentage of cells in the S phase. Untreated cells exhibited the expected pattern for continuously growing cells. More specifically, when the HEK-293 cells were incubated for 12 h with 150 μg mL^−1^ of extract, the percentage of cells at the G_2_/M phase increased by 7 ± 1% compared to the control. Incubation with the extract for 24 h further increased the cells in the G_2_/M phase by 10 ± 1% compared to the control. Similarly, the percentage of HaCaT cells in the G_2_/M phase was found to be increased by nearly 5 ± 0.8% after treatment with 100 μg mL^−1^ for 24 h compared to the control. Results were statistically significant for *p* < 0.01. In all cases, cells treated with the extract present a shift in their cell cycle towards the G_2_/M phase. These results further strengthen the findings of the cell viability assay that the extract promotes cell proliferation and give a better insight into the way that cells proliferate. To the best of our knowledge, this is the first time that the effect of *Urtica dioica* L. extract on the cell cycle progression is examined. Compared to other reports, the effect of our extract on the cell cycle is either similar or better [29,30]. For instance, Li et al. examined the effect of an ethanolic extract of *Periplaneta americana* L. and found that when a solution of 2000 μg mL^−1^ was added to the HaCaT cells it increases their number in the G_2_/M phase. This concentration is twenty times higher compared to the one used in our study [29].

### 2.5. Cell Migration

As can be seen in Figure 2C(A–E), the addition of the extract increased the cell migration rate of HEK-293 and HaCaT cells, compared to control cells, after 24 h (*p* < 0.01). After 24 h the scratch closure observed for control HEK-293 cells was 32 ± 2%. The addition of 150 μg mL^−1^ of the extract caused an almost two-fold increase in the scratch closure (88 ± 4%), while the addition of 100 μg mL^−1^ increased scratch closure by approximately 1.5 times (73 ± 3%). In the case of the positive control, scratch closure was found to be 77 ± 3%. In all cases, samples were statistically different compared to the control for *p* < 0.01. When cells were treated with 100 μg mL^−1^ of extract, no statistically significant difference was recorded between the sample and the positive control, while a statistically significant difference was recorded between the sample and positive control in the case of incubation with 150 μg mL^−1^. In regard to HaCaT cells, scratch closure was found to be 20 ± 2% and 50 ± 3% for control and positive control cells, whereas upon incubation with 100 μg mL^−1^ of the extract, scratch closure was found to be 68 ± 4% (statistically significant difference compared to the control for *p <* 0.01). According to the above results, it can be inferred that the extract promotes cell migration, resulting in faster closure of the scratch area, a benefit that can be reaped to promote wound healing. Cell migration, along with cell proliferation, are pivotal factors in wound healing and many reports have been published aiming to find extracts with cell migration properties [27,31,32]. However, there are reports that exhibit cell migration properties of plant extracts that do not combine cell proliferative properties [27,31]. The fact that the extract presented herein exhibits both properties further highlights its wound healing potential since it can act in more than one of the wound healing stages.

### 2.6. Anti-Inflammatory Activity

#### 2.6.1. Red Blood Cell Membrane Stabilization

**Figure 3:**
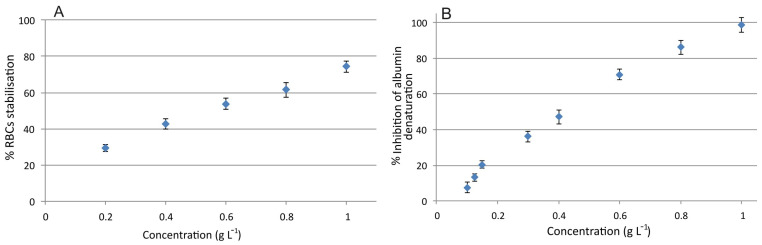
(**A**) Red blood cell membrane stabilization and (**B**) inhibition of albumin denaturation by *Urtica dioica* L. extract.

Inflammation is the physiological response of living tissue to an external stimulus and is part of the wound healing process. During inflammation, lysis of lysosomes occurs, which in turn causes more disorders owing to the released enzymes. Thus, compounds with protective properties against lysosome lysis, such as non-steroidal anti-inflammatory drugs are considered beneficial for wound healing. Due to structural similarities of red blood cells (RBCs) and lysosomes, stabilization of the membrane of RBCs is indicative of anti-inflammatory activity [33]. As can be seen in Figure 3A, a concentration of 1 g L^−1^ stabilizes the membrane of RBCs by nearly 75 ± 4%, compared to control samples. When 0.2 g L^−1^ is used, a 30 ± 2% stabilization is recorded. 

#### 2.6.2. Protein Denaturation Inhibition

Denaturation of proteins has been correlated with inflammation, leading to inflammatory diseases [34]. A substance able to inhibit protein denaturation, therefore, has a potential anti-inflammatory activity. In our case, we examined the potential of the extract to inhibit the heat-induced denaturation of albumin. As it can be seen in Figure 3B the extract can inhibit albumin denaturation at a concentration of 1 g L^−1^, while a 50% inhibition can be achieved by 0.42 g L^−1^. Since the inhibition of protein denaturation at lower concentrations (e.g., 0.1 and 0.2 g L^−1^) is relatively low, it can be inferred that the wound healing potential of the extract is not attributed, mainly, to the anti-inflammatory activity. However, the anti-inflammatory activity of the extract may contribute to the overall process, so that wound healing can be completed in a shorter time. In a previous study, it was evidenced that an aqueous extract of *Urtica dioica* prepared after the sequential extraction of bioactive compounds with other solvents exhibited a lower anti-inflammatory effect on carrageenan-induced hind paw edema in Wistar rats compared to the other fractions (hexane, methanol, ethyl acetate, etc.) [35]. Although our extract is prepared in a similar way and it was expected to exhibit lower anti-inflammatory properties, its effect was comparable or even better compared to previous reports [36,37,38]. For instance, 200 μg mL^−1^ of the methanolic extract of *Mucuna pruriens* reduces the hemolysis of red blood cells by 8.66%, while in our case the percentage was nearly 30% [38].

### 2.7. Antioxidant Activity of the Extract

Among the numerous biological activities that plants possess, their antioxidant activity is well-known. The activity of natural antioxidant compounds depends on many parameters (e.g., matrix complexity, oxidation parameters, oxidation reactions mechanisms, etc.) [39]. In order to gain deep knowledge of the antioxidant activity of a plant extract, many antioxidant assays should be employed. In our case, we conducted a thorough study of the antioxidant properties of *Urtica dioica* L. by employing several antioxidant assays.

#### 2.7.1. DPPH Assay

**Figure 4:**
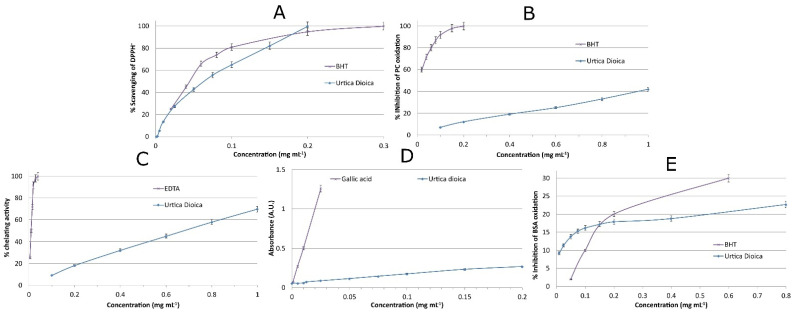
(**A**) Free radical scavenging, (**B**) inhibition of lipid oxidation, (**C**) metal chelating, (**D**) reducing power, and (**E**) prevention of protein oxidation properties of *Urtica dioica* L. extract.

It has been reported that there are many free radicals in wound sites that cause oxidative stress, resulting in DNA, protein, cell membrane, and lipid damages, resulting in cytotoxicity and delayed wound healing [6,40]. Therefore, it is conceived that free radical scavenger compounds can facilitate the overall wound healing process [6]. The first step of our study was to examine the potential of the extract to scavenge DPPH free radicals. As can be seen in Figure 4A, the scavenging activity of the extract is similar to that of butylated hydroxytoluene (BHT), a well-known antioxidant compound. Total DPPH scavenging was achieved using 0.2 g L^−1^ of the extract. This suggests that the concentration used for the in vivo experiments can scavenge free radicals, among others, and assist with the wound healing process.

#### 2.7.2. Inhibition of Lipid Oxidation—TBARS Assay

Lipid peroxidation results in damages to endothelial and fibroblast cells, alters keratinocyte capillary permeability, and reduces collagen metabolism [40]. Thus, it was suggested that compounds with lipid peroxidation inhibition properties can enhance collagen synthesis and protect fibroblasts. The *Urtica dioica* L. extract examined herein is not able to inhibit phosphatidylcholine oxidation to a considerable degree, compared to BHT (Figure 4B). A concentration of 0.2 g L^−1^ of the extract can inhibit phosphatidylcholine oxidation by 12 ± 1%. Although the percentage is relatively low, it may have a contribution to the overall antioxidant protection of the cells in the wound site.

#### 2.7.3. Metal Chelating Activity

Oxidative damage in wound sites, occurring, mainly, in macrophages and neutrophils, can take place by Fenton reactions [41]. It is reported that iron-catalyzed oxidative stress leads to faster apoptosis and, thus, wound healing time increases. As can be seen from Figure 4C, the extract can achieve up to 70 ± 2% chelating activity when 1 g L^−1^ was used, while a concentration of 0.2 g L^−1^ has ~20% chelating activity. Although these percentages are far lower than that of ethylenediaminetetraacetic acid (EDTA), a strong chelating agent, they are not considered negligible for a natural product [42]. Another benefit that can occur from the chelating activity of the plant extract, aside from the inhibition of Fenton-reaction-based oxidative stress, is the control of bacterial infections. Iron, which is essential for bacterial growth, is withdrawn from the wound site by the chelating agents of the extract, and bacteriostasis can occur. This is the same mechanism of action as the lactoferin protein [43].

#### 2.7.4. Reducing Power

It is reported that the reducing power of plant extracts is directly associated with their antioxidant activity [42]. With regard to the reducing power of the extract, the electron donor ability of the compounds contained in the extract is limited compared to that of BHT (Figure 4D). The electron donor ability is reported to be the main mechanism of the antioxidant activity of phenolic compounds [43]. Therefore, it is speculated that the contribution of phenolic compounds to the overall antioxidant activity is low. 

#### 2.7.5. Prevention of Oxidative Damage to Proteins

Proteins are essential parts of living organisms. Oxidative stress in wound areas can impair proteins and their functionality, leading to cell apoptosis and senescence [44]. According to our results, the extract has a moderate potential to prevent oxidative damage to proteins, as shown in Figure 4E. In addition, BHT has a similar protective action against protein oxidation. Taking into consideration that several plant extracts or pure compounds, with known antioxidant properties, can barely protect proteins from metal-catalyzed oxidation, the antioxidant potential of *Urtica dioica* L. extract is highlighted [42].

### 2.8. Blood Compatibility

As stated elsewhere [45], before conducting experiments in animal models, it is prudent to examine the blood compatibility of the tested compound. Despite the beneficial effect of a tested substance, if it is not compatible with blood (and other constituents of living organisms) then health risks arise. Results from the blood compatibility assay revealed that no red blood cell lysis occurs after their incubation with up to 1 g L^−1^ of the extract. Moreover, no effect on the blood clot time was observed. Therefore, the extract can be safely administered to the animals for in vivo experiments.

### 2.9. In Vivo Wound Healing Experiments

#### 2.9.1. Wound Closure

**Figure 5:**
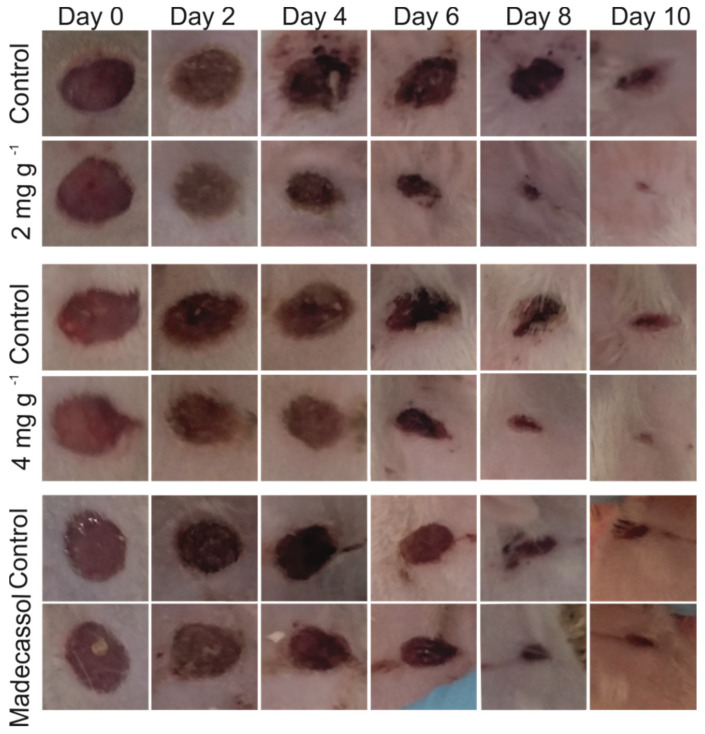
Representative photographs of wounds at days 0, 2, 4, 6, 8 and 10 from rats treated with 2 and 4 mg g^−1^ of *Urtica dioica* L. extract and Madecassol.

All the aforementioned in vitro experiments corroborate that the *Urtica dioica* L. extract is suitable for wound healing. This is because it can assist the overall process in more than one stage. To validate our findings, we conducted wound healing experiments in rat models. Rats were treated with two concentrations of the extract (i.e., 200 and 400 μg per dose). Wounds treated with the extract showed a significant difference in terms of wound closure compared to the control groups from the second day onwards. Representative photos of control and extract-treated wounds at days 0, 2, 4, 6, 8, and 10 can be seen in Figure 5.

As can be seen, wound closure was accelerated when wounds were treated with both concentrations of the extract, although better results were recorded at the lower tested concentration. Wound closure between treated and non-treated wounds was statistically significant for *p* < 0.01. Moreover, in the control wound, persistent inflammation was apparent by macroscopic observation for several days, while no signs of inflammation could be observed in the wounds treated with the extract from day 2. This is in accordance with the above-discussed findings: that the extract has anti-inflammatory properties. By applying the ointment, wounds were healed in a shorter period (9–10 days) compared to control wounds that were treated in 13 days. It is noteworthy that the biggest difference between the control and the treated wounds was observed on day 6. This is due to the enhancement of cell proliferation and migration in the presence of the extract, two processes that occur more actively at the third stage of wound healing.

**Figure 6:**
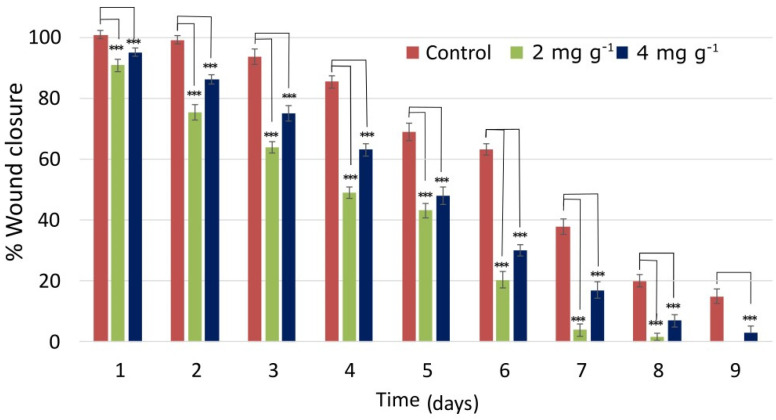
Graphical representation of the % wound area (compared to day 0) at different days after wound creation upon treatment with 2 and 4 mg g^−1^ of *Urtica dioica* L. extract; statistically significant differences for *p* < 0.001 are denoted with ***.

When compared to the positive control (Madecassol cream), better results were recorded in the case of the extract. At days 2 and 4, the area of the wound treated with Madecassol was reduced by 6 ± 1% and 14 ± 2%, respectively, while the area of the wound treated with 2 mg g^−1^ of the extract was reduced by 23 ± 1.5% and 50 ± 2.5%, respectively (Figure 6). Similarly, on day 6, the area of the Madecassol-treated wound was reduced by 45 ± 2% and the area of the *Urtica*-*dioica*-L.-extract-treated wound was reduced by 80 ± 2.5%. The results concerning the wound healing potential of Madecassol cream are in accordance with previous reports [46,47]. Finally, the wound treated with Madecassol fully healed on day 12, while *Urticadioica* L. extract-treated wounds healed between days 9 and 10.

In a study by Razika et al., the authors prepared a saponin extract from Algerian *Urtica dioica* L. and examined its wound healing potential [19]. The authors found that the prepared saponin extracts were able to scavenge the DPPH free radicals (500 μg mL^−1^ of the extract was needed to achieve ~90% scavenging), and based solely on this property they exhibited its wound healing properties. They prepared a formulation containing 20% *w*/*w* of the extracts and applied it to wounds on rats. According to their results, no statistically significant wound healing properties could be observed until day 7, while the effect was more pronounced later. Compared to this extract from *Urtica dioica* L., our extract is superior, since it combines cell proliferation, cell migration, antioxidant, and anti-inflammatory properties. Moreover, with a much smaller amount of the extract (0.2% *w*/*w*), its effect on wound healing was apparent from the first day of application. In the study of Bouassida et al., the antioxidant and antibacterial properties of a hydroethanolic extract from *Urtica dioica* L. were presented along with its wound healing properties [18]. When the extract was applied to the wounds, higher wound contraction was observed in the experimental group after day 5 and until day 11, which, however, was not higher than 20% compared to the control group. The above studies with extracts from the same plant highlight the potential of our extract for further applications, since it mounts many different actions, making it more efficient at lower concentrations. 

#### 2.9.2. Histopathological Study

**Figure 7:**
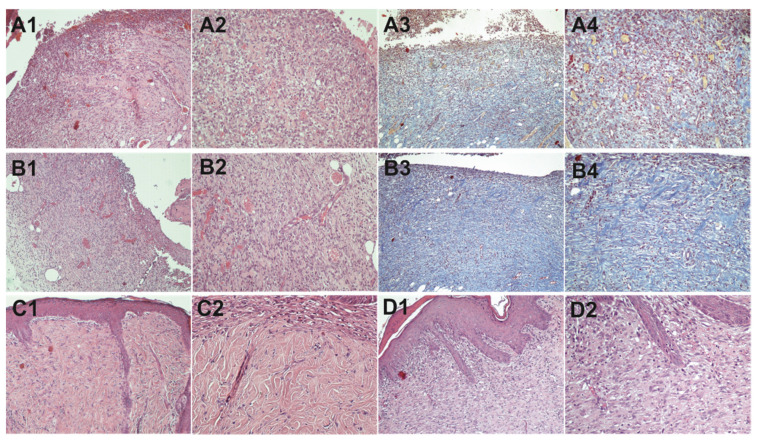
Tissue images of control wounds at day 5 (**A1**–**A4**) and day 10 (**C1**,**C2**) and *Urtica dioica* L. ointment (2 mg g^−1^)-treated wounds at day 5 (**B1**–**B4**) and day 10 (**D1**,**D2**) at 100× (odd-numbered images) and 200× magnification (even-numbered images). All tissues were stained with hematoxylin and eosin, except for images **A3**, **A4**, **B3**, and **B4**, which show tissues stained with Masson’s trichrome.

In the control wound at day 5, vascular inflammatory granulomatous tissues with abundant inflammatory cells and thin-walled capillary blood vessels with stimulated endothelial cells within an edematous layer are observed (Figure 7). Moreover, few fibroblasts are observed, and maturation begins at the deeper sites, while re-epithelization begins in the periphery. The photographs of the test wound tissue at day 5 show that the inflammatory granulomatous tissue is more mature with fewer chronic inflammation cells. Additionally, significantly reduced edema and capillary blood vessels with more mature/flattened endothelial cells can be observed. Additionally, most capillary blood vessels appear to be perpendicular to the overlying epidermis. Finally, as evidenced by Masson’s trichrome staining, fibroblasts are increased both at superficial and deep positions. In both tissue samples at day 10, complete re-epithelialization is observed with concomitant growth of fibrous connective tissue in the dermis, while the two samples exhibit many differences. In the extract-treated wounds, dense connective tissue with a markedly developed capillary network and more fibroblasts compared to non-treated wounds can be seen. The granular tissue and fibroblasts are more mature, and the wound is covered with more keratinocytes in the case of extract-treated wounds. In the control wound, only a few layers of immature keratinocytes can be observed, the connective tissue is not so dense, and a small number of fibroblasts and immature capillaries can be seen. It is noteworthy that in the control sample, scar tissue is visible, while in the extract-treated sample the wound area is completely re-established. Finally, in the basal layer of the extract-treated sample, more folds are observed, which are indicative of increased strength of the new tissue. Owing to all the above, it is evident that *Urtica dioica* L. accelerated the wound healing process.

For the preparation of a well-grounded formulation for topical application, more research is needed. Further fractionation of the extract could probably be carried out to simplify the composition of the formulation, but without taking a toll on its efficiency. Moreover, further studies could be carried out to further elucidate the performance of other components in wound healing, such as immunohistochemical analyses to study proteins. Additionally, more properties of the formulations should be studied, such as the occlusive effect and the ability to permeate the skin. 

## 3. Materials and Methods

### 3.1. Cell Lines and Chemicals

Human embryonic kidney 293 (HEK-293) cells and immortalized human keratinocytes (HaCaT) (obtained from ATCC (Manassas, VA, USA)) were used for the in vitro experiments. Unless otherwise specified, all reagents were purchased from Sigma Aldrich (Steinheim, Germany). Fetal bovine serum, RPMI-1640, DMEM (high-glucose), penicillin, and streptomycin were purchased from Gibco. Propidium iodide was purchased from Thermo Fischer Scientific (Waltham, MA, USA). Chemco hydrophilic base aqueous cream and Madecassol cream was bought from a local pharmacy store (Ioannina, Epirus, Greece).

### 3.2. Plant Material and Extract Preparation

Aerial parts of *Urtica dioica* (subspecies *dioica* L.) were purchased from a local store, (Ioannina, Epirus, Greece). The sample was identified and reference specimens were retained in the University of Ioannina with voucher accession number UOI 120915. Plant parts were kept at −20 °C until use.

A portion of 50 g of *Urtica dioica* L. was powdered using liquid nitrogen. The constituents of the pulverized plant were extracted via Soxhlet extraction using ethyl acetate for 3 h, followed by extraction with acetonitrile for 3 h more. After extraction, the plant was boiled in double-distilled water for 5 min and filtered through a grade 4 Whatman filter paper. Water was removed by freeze-drying and a powder-like solid was obtained. The dry extract was stored in amber glass vials at −80 °C to avoid potential chemical degradation. For in vivo studies, 0.20% or 0.40% (*w*/*w*) ointment was prepared by mixing the extract in Chemco hydrophilic base aqueous cream.

### 3.3. Phytochemical Analysis and NMR Fingerprinting

The ^1^H NMR fingerprint of the extract was recorded using a Bruker AV-500 MHz instrument (Karlsruhe, Germany). The extract was dissolved in D_2_O (5 mg mL^−1^) and the NMR spectra were recorded at 298 ± 1 K. The one-dimensional spectrum was acquired with a sweep width of 6000 Hz (12 ppm), 32 K points for the free induction decay, an acquisition time of 3.4 s, a relaxation delay of 4.6 s, and 256 scans. Water suppression was achieved using the WATERGATE-5 sequence. Prior to Fourier transform, exponential window function (with line broadening 0.3 Hz) was applied to the free induction decay, to improve the signal-to-noise ratio. For the identification of specific groups of phytochemicals in the extract, qualitative screening experiments were performed. Analyses were carried out according to previously reported methods [48,49].

#### 3.3.1. Test for Amino Acids

In 1 mL of extract solution, ninhydrin was added, and the mixture was boiled for a few minutes. Appearance of a blue color is indicative of the presence of amino acids.

#### 3.3.2. Test for Xanthoproteins

In 1 mL of extract solution, a few drops of conc. HNO_3_ or conc. NH_3_ were added. Appearance of a reddish–orange precipitate indicated the presence of xanthoproteins.

#### 3.3.3. Test for Quinones

In 1 mL of plant extract solution, a few drops of alcoholic potassium hydroxide solution were added. Appearance of a red-to-blue color indicated the presence of quinones.

#### 3.3.4. Test for Coumarins

In a test tube a portion of the extract was transferred, and 1–2 drops of water were added to moisten it. A filter paper treated with 1 mol L^−1^ sodium hydroxide solution was used to cover the mouth of the tube and then the tube was placed in a boiling water bath for a few minutes. The filter paper was removed and examined under UV light. Appearance of a yellow fluorescence indicated the presence of coumarins.

#### 3.3.5. Test for Carboxylic Acids

In 1 mL of extract solution, a few drops of sodium bicarbonate solution were added. Effervescence is indicative of carboxylic acids.

#### 3.3.6. Test for Tannins

In 1 mL of extract solution, a few drops of 1% (*w*/*v*) ferric chloride solution were added. Appearance of an intense blue, black, green, or purple color suggests the presence of tannins.

#### 3.3.7. Test for Glycosides

In a test tube, five mL of extract solution were transferred and hydrolyzed by adding 5 mL conc. HCl and boiling the mixture for a few hours. A small amount of the hydrolyzed extract was dissolved in 1 mL of water and then a small amount of 10% (*w/v*) sodium hydroxide was added. Appearance of a yellow color indicated the presence of glycosides.

#### 3.3.8. Test for Flavonoids

Extract (defatted previously with petroleum ether) was dissolved in 80% *v*/*v* ethanol solution and 3 mL was transferred to a test tube. Appearance of a yellow color, after the addition of 4 mL (1% *w*/*v*) of potassium hydroxide, is indicative of the presence of flavonoids.

#### 3.3.9. Test for Saponins

Extract solution was vigorously shaken. Appearance of a froth, stable for at least 1 min, is indicative of the presence of saponins.

#### 3.3.10. Test for Terpenoids

In a test tube, 5 mL of extract solution was added, followed by the addition of 2 mL of chloroform. After slowly adding 3 mL of conc. H_2_SO_4_, the color of the interface was observed. Appearance of a red–brown color suggested the presence of terpenoids.

#### 3.3.11. Test for Cardiac Glycosides

In 2 mL of extract solution, 1 mL of glacial acetic acid was added, followed by the addition of 1 mL of ferric chloride solution and 1 mL of conc. H_2_SO_4_. Green or blue coloration of the solution indicated the presence of cardiac glycosides.

#### 3.3.12. Test for Resins

In 1 mL of extract solution, a few drops of acetic anhydride solution were added, followed by the addition of 1 mL of conc. H_2_SO_4_. Appearance of an orange–yellow color indicated the presence of resins.

#### 3.3.13. Test for Steroids

In 2 mL of acetic anhydride a small portion of extract was added and then 2 mL of conc. H_2_SO_4_ was added. A change in color from violet to blue or green is indicative of the presence of steroids.

#### 3.3.14. Test for Phenols

In 1 mL of extract solution, 2 mL of water and a few drops of 10% (*w*/*v*) ferric chloride solution were added. Appearance of a blue or green color indicated the presence pf phenols.

#### 3.3.15. Test for Reducing Sugars

To 5 mL of extract solution, 25 mL of diluted H_2_SO_4_ solution was added and the mixture was boiled for 15 min. After cooling, the pH was adjusted to 7 using 10% (*w*/*v*) sodium hydroxide solution, and 5 mL of Fehling solution was then added. The formation of a deep-red precipitate indicated the presence of reducing sugars. Alternatively, equivolume amounts of plant extract solution and Benedict’s reagent were mixed and placed in a boiling water bath for 5 min. Appearance of a green, yellow, or red color indicated the presence of reducing sugars and depended on their amount.

#### 3.3.16. Test for Anthraquinones

In a test tube, a portion of the extract was added along with 1 mL of 10% (*w*/*v*) ferric chloride solution and 1 mL of conc. HCl; the mixture was heated. After cooling, an equal amount of chloroform was added, and the mixture was shaken for 1 min. To 2 mL of the chloroform phase 1 mL of 10% (*v*/*v*) ammonia solution was added. A deep-red or pink color of the aqueous phase indicated the presence of anthraquinones.

#### 3.3.17. Test for Phlobatannins

In a test tube, 2 mL of extract solution and 2 mL of 1% (*v*/*v*) conc. HCl were transferred, and the mixture was boiled for a few minutes. Formation of a red precipitate indicated the presence of phlobatannins.

#### 3.3.18. Test for Proteins

In 1 mL of extract solution, 1–2 drops of conc. HNO_3_ were added. Appearance of a yellow color indicated the presence of proteins. Alternatively, to 3 mL of plant extract solution, a few drops of 4% (*w*/*v*) sodium hydroxide solution were added, followed by the addition of a few drops of 1% (*w*/*v*) copper sulphate solution. Appearance of a pink color indicated the presence of proteins.

#### 3.3.19. Test for Carbohydrates

In 5 mL of extract solution, 2 drops of an alcoholic 1-naphthol solution were added. Formation of a violet ring at the interface indicated the presence of carbohydrates.

#### 3.3.20. Test for Monosaccharides

Equivolume amounts of extract solution and Barfoed’s reagent (i.e., 0.33 mol L^−1^ neutral copper acetate in 1% acetic acid) were mixed and heated for 2 min in a boiling water bath. Formation of a red precipitate indicated the presence of monosaccharides.

#### 3.3.21. Test for Aldoses/Ketoses

To 1 mL extract solution, 5 mL of Seliwanoff’s reagent (i.e., resorcinol and conc. HCl) was added and the mixture was placed in a boiling water bath for 5 min. Formation of a red color indicated the presence of ketoses.

### 3.4. In Vitro Experiments

Cell cultures, a cell proliferation assay, a cell migration study, and cell cycle analysis were carried out according to our previous study [45]. 

#### 3.4.1. Cell Proliferation Assay

HEK-293 cells were grown in a RPMI-1640 growth medium and HaCaT cells were grown in a DMEM high-glucose medium. Cell proliferation was evaluated using the crystal violet assay. In brief, an aliquot of an aqueous extract solution at a proper concentration was added to fresh growth medium prior to the addition of the cultured cells, resulting in different final concentrations of the extract (i.e., 5, 50, 100, and 150 μg mL^−1^) in each well; cells were further incubated for 24 h. For each condition, three identical samples were prepared. Cells that were incubated in the absence of the plant extract were used as controls. Cell viability was assessed employing the crystal violet assay and was calculated as a percentage of control cells using the following Equation (1):

(1) $$\mathrm{cell}\;\mathrm{viability}\;\left(\%\right)=\left(\frac{{\mathrm{OD}}_{\mathrm{treated}}}{{\mathrm{OD}}_{\mathrm{control}}}\right)\times 100$$

where OD_control_ is the average absorbance of the non-treated cells and OD_treated_ is the average absorbance of the treated cells. All results are expressed compared to control.

#### 3.4.2. Cell Cycle Analysis

To analyze cell cycle progression, HEK-293 and HaCaT cells were first synchronized with serum starvation for 20 h. After serum starvation, a high percentage of cells were inhibited at the G_0_/G_1_ phase. Cells were restimulated for 24 h, in order to enter the cell cycle, by adding 10% serum. Then, cells were seeded into 24-well plates, where 100 or 150 μg mL^−1^ of the extract was added and incubated for 12 or 24 h. Cells were then trypsinized, collected in centrifuge tubes and centrifuged at 2200 rpm for 6 min, at room temperature. The supernatant was rejected, and the resulting cell pellet was washed with PBS, and centrifuged to the above conditions. The washing step was repeated once more. After discarding PBS, cells were fixed by adding, dropwise, 4.5 mL of 70% (*v*/*v*) cold ethanol to the cell pellet and being stored at −20 °C overnight. The cell suspension was centrifuged at 2200 rpm for 6 min, ethanol was removed, and the resulting cell pellet was washed twice with PBS according to the aforementioned procedure. The cell pellet was resuspended in propidium iodide solution, incubated at 37 °C in the dark for 45 min, and then analyzed by fluorescence-activated cell sorting (FACS) on a FACS scan flow cytometer (BD FACS Aria) for relative DNA content, based on red fluorescence. A minimum of 15,000 events were recorded for each sample and the proportion of cells in the G_0_/G_1_, S, and G_2_/M phases of the cell cycle, was calculated from the resulting DNA histograms using a planimetrie method of analysis with BD FACS Diva software V9.0 (BD Biosciences, Franklin Lakes, NJ, USA).

#### 3.4.3. Cell Migration Assay

In brief, an elongated wound was generated on a monolayer of cells using a sterile 200 μL tip to create an approximately wide scratch. Plant extract was applied to the cells at different concentrations. Cells not treated with the extract were taken as controls. Photographs were taken at a 5× magnification using a microcamera with an inverted phase microscope (Olympus CK40) (Olympus Optical Co. (EUROPA) GMBH, Hamburg, Germany), after 24 h of incubation. The images acquired for each sample were further analyzed using Leica LAS X software. The average size of the gap was calculated using measurements from three different points for each sample. Wound closure (%) was calculated using the following Equation (2):

(2) $$\mathrm{Wound}\;\mathrm{closure}\left(\%\right)=\frac{{\mathrm{ADBC}}_{0}-{\mathrm{ADBC}}_{t}}{{\mathrm{ADBC}}_{0}}\times 100$$

where ADBC stands for the average distance between the scratch at time 0 (ADBC_0_) and the different time points (ADBCt).

### 3.5. Anti-Inflammatory Properties

Inhibition of albumin denaturation and stabilization of the membrane of human red blood cells were studied following previous reports [34,50].

#### 3.5.1. Inhibition of Albumin Denaturation

In a reaction vessel 1 mL of PBS, 1 mL of bovine albumin fraction solution (1% *w*/*v* in PBS), and 1 mL of plant extract solution (of appropriate concentration), prepared in PBS, were added. Samples were incubated at 37 °C for 20 min and then heated at 70 °C, for 20 min. After cooling to room temperature, the turbidity was measured spectrophotometrically at 660 nm. For each tested concentration triplicates were prepared. Inhibition of protein denaturation (%) was calculated using the following Equation (3):

(3) $$\mathrm{Inhibition}\;\left(\%\right)=\frac{{\mathrm{ABS}}_{\mathrm{control}}-{\mathrm{ABS}}_{\mathrm{sample}}}{{\mathrm{ABS}}_{\mathrm{control}}}\times 100$$

where ABS_control_ and ABS_sample_ denote the turbidity of the control and sample, respectively.

#### 3.5.2. Human Red Blood Cell Membrane Stabilization

In brief, a volume of human red blood cell solution was mixed with an equal volume of plant extract solution (so that the final concentration ranged between 0.2–1.0 mg mL^−1^) and the mixture was incubated at 56 °C for 30 min. After centrifugation, the absorbance was measured at 560 nm. The red blood cell membrane stabilization was calculated using the following Equation (4):

(4) $$\mathrm{RBCs}\;\mathrm{Stabilization}\;\left(\%\right)=\frac{{\mathrm{ABS}}_{\mathrm{control}}-{\mathrm{ABS}}_{\mathrm{sample}}}{{\mathrm{ABS}}_{\mathrm{control}}}\times 100$$

where ABS_control_ and ABS_sample_ were the absorbance measurements of the control and sample, respectively.

### 3.6. Antioxidant Assays

DPPH free radical scavenging, inhibition of lipid peroxidation, metal chelating activity, reducing power and prevention of oxidative damage to proteins were examined following previously reported methods [42,51].

#### 3.6.1. DPPH Assay

In brief, 3.9 mL of DPPH solution (1:2 methanol:water, 6 × 10^−5^ mol L^−1^) was mixed with 1.1 mL of a methanol:water mixture (1:2) containing the extract at various concentrations, and the mixture was stirred for 30 min at room temperature. The absorbance of the solution was measured at 515 nm. As a blank experiment, pure methanol was added to the DPPH solution. Free radical scavenging activity (%) was determined using the following Equation (5):

(5) $$\%\;\mathrm{Scavenging}\;\mathrm{of}\;\mathrm{DPPH}=\frac{A_{o}-A}{A_{o}}\times 100$$

where *A_o_* and *A* are the absorbances of the blank solution and the sample, respectively.

#### 3.6.2. Inhibition of Lipid Oxidation—TBARS Assay

In a test tube, 40 μL of phosphatidylcholine (PC) solution (10 mg mL^−1^ in PBS), 60 μL of KCl (1 mol L^−1^), 80 μL of FeCl_3_ (0.001 mol L^−1^), 200 μL of water, containing the extract at various concentrations, and 20 μL of ascorbic acid (0.001 mol L^−1^) were mixed and incubated for 1 h at 37°C. Then, 10 μL of butylated hydroxytoluene (BHT) solution 0.002% (*w*/*v*) in methanol, 400 μL of trichloroacetic acid 2.8% (*w*/*v*), and 400 μL of thiobarbituric acid 1.0% (*w*/*v*) in 0.05 mol L^−1^ NaOH were added successively, and the mixture was incubated at 100 °C, for 20 min. After cooling for 5 min in an ice bath, 1 mL of *n*-butanol was added, and the mixture was shaken vigorously. After centrifugation for 5 min at 3000 rpm, the absorbance of the organic layer was measured at 532 nm. Lipid oxidation inhibition (%) of PC was calculated using the following Equation (6):

(6) $$\%\;\mathrm{Inhibition}\;\mathrm{of}\;\mathrm{PC}\;\mathrm{oxidation}=\frac{A_{o}-A}{A_{o}}\times 100$$

where *A_o_* and *A* are the absorbance of the blank solution (in the absence plant extract) and the sample, respectively.

#### 3.6.3. Metal Chelating Activity

In a test tube, 3 mL of aqueous plant extract solution (of proper concentration), 3.7 mL of water, 0.1 mL of FeCl_2_ (0.002 mol L^−1^) and 0.2 mL of ferrozine (0.005 mol L^−1^) were added and the mixture was stirred for 10 min. After 10 min, the absorbance was measured at 562 nm. Metal chelating activity (%) (i.e., inhibition of the formation of the ferrozine–Fe^2+^ complex) was calculated using the following Equation (7):

(7) $$\%\;\mathrm{Metal}\;\mathrm{chelating}\;\mathrm{activity}=\frac{A_{o}-A}{A_{o}}\times 100$$

where *A_o_* and *A* are the absorbance of the blank solution (in the absence plant extract) and the sample, respectively.

#### 3.6.4. Reducing Power 

In a test tube, 2.5 mL of aqueous plant extract solution (of proper concentration), 2.5 mL of phosphate-buffered solution (pH 6.6, 0.2 mol L^−1^), and 2.5 mL of K_3_[Fe(CN)_6_] 1% (*w*/*v*) were added, mixed, and incubated at 50 °C for 20 min. Then, 2.5 mL of trichloroacetic acid (10% *w*/*v*) solution was added, and the mixture was centrifuged at 3000 rpm for 5 min. An aliquot of 2.5 mL was retracted from the supernatant and transferred into another test tube, where 2.5 mL of distilled water and 0.5 mL of FeCl_3_ (0.1% *w*/*v*) were added. After 10 min of stirring, the absorbance was measured at 700 nm. The blank solution consisted of water, without the addition of plant extract.

#### 3.6.5. Prevention of Oxidative Damage to Proteins

Prevention of oxidative damage to proteins was based on a Fenton/bovine serum albumin (BSA) reaction. In brief, 100 μL of BSA solution (20 mg mL^−1^ in PBS), 100 μL of FeCl_2_/citric acid solution (0.0044/0.0040 mol L^−1^ in water), 100 μL of H_2_O_2_ (0.0044 mol L^−1^), and 100 μL of aqueous plant extract solution were added and the mixture was incubated at 37 °C for 1 h. After the addition of 1 mL of 2,4-dinitrophenyl-hydrazine (0.01 mol L^−1^ in HCL (2 mol L^−1^)) the mixture was further incubated for 1 h, with periodic shaking every 15 min. Then, 1 mL of trichloroacetic acid solution (20% *w*/*v*) was added, and the mixture was centrifuged at 3000 rpm for 5 min. The supernatant was decanted and the precipitate was washed three times with 2 mL of ethanol:ethyl acetate (1:1 *v*/*v*). Finally, the precipitate was dissolved in 1.2 mL of guanidine aqueous solution (6 mol L^−1^) and the mixture was incubated at 37 °C, for 20 min. After centrifugation, the absorbance was measured at 390 nm. A blank sample was prepared by adding water.

### 3.7. Blood Compatibility

Blood compatibility of the extract was examined following a previously reported procedure [45]. In brief, human blood was collected and mixed with an equal volume of normal saline (3% *w*/*v* sodium citrate was added as a coagulant in the blood prior to mixing). In 1 mL of blood, normal saline containing the extract at various concentrations was added and the mixture was incubated for 60 min at 37 °C. Then, the samples were centrifuged and the optical density at 540 nm was recorded. Normal saline and 1% Triton-X were used as negative and positive controls, respectively.

### 3.8. Study of the Wound Healing Efficacy

#### 3.8.1. Animals

A total of 24 Wistar male rats weighing 200–220 gr were used. Rats were housed in individual cages in laboratory conditions under an ambient temperature and a 12 h dark and light cycle. Water and food were provided to the animals ad libitum. Experiments were conducted in accordance with the internationally accepted principles for laboratory animal use and care (EU Directive of 2010/63/EU for animal experiments) and approved by the institutional animal ethical committee (University of Ioannina, Greece, protocol no. 2757/22.03.2019).

#### 3.8.2. Full-Thickness Wound Creation

Rats were anesthetized by a ketamine–xylazine injection (80 mg kg^−1^:10 mg kg^−1^) intraperitoneally. Under anesthesia, the dorsal skin was shaved and disinfected using 70% alcohol, while antisepsis was achieved using betadine. On both sides of the depilated dorsum of each rat, a full-thickness dorsal wound was created using an 8 mm skin biopsy punch (area of wound: 50 mm^2^) [52].

#### 3.8.3. Treatment Schedule

In each rat, one wound served as control and the other for testing reasons. Each rat received a single dose of ointment (i.e., 100 mg of Chemco hydrophilic base aqueous cream containing 0.20% or 0.40% (*w*/*w*)) once a day. The dose was selected so that the amount of the extract applied could be the same as that of the in vitro studies. Animals were divided into 3 groups (6 rats in each group). Two groups were treated with the ointment of *Urtica dioica* L. and the third group was treated with Madecassol, a commercially available formulation used for wound healing, which served as a positive control. The rest of the animals were used for the histopathological study. The skin of all rats was inspected on a daily basis and wounds were photographed from a fixed distance. Areas of the wounds were calculated from each photo using the Image J software. More specifically, for each photo, the image scale was first set and then photos were converted into grayscale. After properly enhancing edges and sharpening the photos, the threshold was automatically calculated and the edges were detected. The area enclosed by the edges was finally calculated. Wound closure percentage was calculated using the following Equation (8):

(8) $$\%\;\mathrm{Wound}\;\mathrm{closure}=\frac{A_{o}-A}{A_{o}}\times 100$$

where *A_o_* was the wound area at the beginning (day 0) and *A* was the wound area at the specified day.

#### 3.8.4. Histopathological Examination

At the end of day 5 and day 10, animals were euthanized and wound tissue specimens from control and test wounds were taken. Tissues were fixed with 10% neutral formalin solution and embedded in paraffin. Using a microtome, 5-μm-thick sections were cut, stained with hematoxylin and eosin or Masson’s trichrome and examined under an optical microscope.

### 3.9. Statistical Analysis

All assays were repeated three times to ensure reproducibility. *p* values lower than 0.05 were considered statistically significant. Results were expressed as mean ± standard deviation. Cell migration assay data were analyzed by a two-way ANOVA with the independent factors being experiment and treatment followed by Duncan’s multiple range test post hoc tests. For the rest of the assays, the levels of significance between samples were compared by one-way ANOVA using Student’s *t*-test as a post hoc test for comparing means. 

## 4. Conclusions

In this study, the effect of *Urtica dioica* L. aqueous extract on two epithelial cell lines was examined so as to highlight its potential for dermal and kidney injuries. It was found that the tested extract can enhance cell proliferation of HEK-293 and HaCaT cells significantly. This effect is achieved via the faster progression of cells in the G_2_/M phase, by completing in a shorter period of time all processes occurring in the S phase, and thus the cell cycle is completed sooner for cells incubated with the plant extract. In addition, the migration rate of cells incubated with the extract was two times higher than that of control cells. Moreover, the plant extract was found to possess significant anti-inflammatory and multifaceted antioxidant properties, owing to which the protection of cells, in the wound site, via various mechanisms is achieved using the extract. Experiments in animal models clearly demonstrated the potential of the extract for wound healing applications. Wounds treated with the extract healed 3–4 days faster compared to untreated wounds and 2 days faster compared to a commercially available formulation, while the new tissue in the case of the extract-treated wound exhibits better characteristics according to the histopathological study compared to the non-treated wound tissue. Overall, it can be concluded that in vivo wound healing is achieved primarily by the cell proliferation, cell migration, and anti-inflammatory activities of the extract, while the overall activity is strengthened by secondary mechanisms achieved by the antioxidant properties of the extract. The above showcase the wound healing potential of an aqueous extract of *Urtica dioica* L. 

## Data Availability

Not applicable.

## Sample Availability

Plant reference specimens are retained in the University of Ioannina with the voucher accession number UOI 120915.
